# 50 Years of Terrorism against the Nuclear Industry: A Review of 91 Incidents in the Global Terrorism Database

**DOI:** 10.1017/S1049023X2300002X

**Published:** 2023-04

**Authors:** Harald De Cauwer, Dennis G. Barten, Derrick Tin, Luc J. Mortelmans, Gregory R. Ciottone, Francis Somville

**Affiliations:** 1. Department of Neurology, Sint-Dimpna Regional Hospital, Geel, Belgium; Faculty of Medicine and Health Sciences, University of Antwerp, Wilrijk, Belgium; 2. Department of Emergency Medicine, VieCuri Medical Center, Venlo, the Netherlands; 3. Department of Emergency Medicine, Beth Israel Deaconess Medical Center, Boston, Massachusetts USA; Harvard Medical School, Boston, Massachusetts USA; 4. Center for Research and Education in Emergency Care, University of Leuven, Leuven, Belgium; REGEDIM, Free University Brussels, Belgium; Department of Emergency Medicine, ZNA Camp Stuivenberg, Antwerp, Belgium; 5. Director, BIDMC Disaster Medicine, Beth Israel Deaconess Medical Center; Associate Professor, Harvard Medical School, Boston, Massachusetts USA; 6. Department of Emergency Medicine, Ziekenhuis Geel, Geel, Belgium; Faculty of Medicine and Health Sciences, University of Antwerp, Wilrijk, Belgium; Faculty of Medicine, University of Leuven, Leuven, Belgium; CREEC (Center for Research and Education in Emergency Care), University of Leuven, Leuven, Belgium

**Keywords:** Counter-Terrorism Medicine, nuclear industry, terrorism, uranium

## Abstract

**Background:**

The on-going Russo-Ukrainian war has resulted in a renewed global interest in the safety and security of nuclear installations and the possibility of nuclear disasters caused by warfare and terrorism.

The objective of this study was to identify and characterize all documented terrorist attacks against nuclear transport, nuclear facilities, and nuclear scientists as reported to the Global Terrorism Database (GTD) over a 50-year period.

**Methods:**

The GTD was searched for all terrorist attacks against nuclear facilities, nuclear scientists, nuclear transport, and other nuclear industry-related targets in the period from 1970-2020. Analyses were performed on temporal factors, location, target type, attack and weapon type, perpetrator type, number of casualties, and property value loss.

**Results:**

Ninety-one incidents that occurred from 1970 through 2020 were included. Incidents took place in 25 countries and nine world regions, with most (42; 46.1%) occurring in Western Europe.

During these 50 years, 91 incidents resulted in 19 fatalities and 117 injuries. One perpetrator was killed during an incident and one other assailant was injured.

Bombings and explosions were the most frequently identified attack type (n = 40; 44.0%), followed by facility/infrastructure damage (n = 24; 26.4%) and armed assaults and assassinations (both n = 7; 7.7%).

Nuclear power plants and reactors under construction were targeted in 13 (14.3%) and eight (8.8%) incidents, respectively. Most of the attacks took place on other nuclear industry-related sites.

**Conclusion:**

Terrorist attacks carried out by non-state perpetrators against nuclear facilities, nuclear scientists, nuclear transport, and other nuclear industry-related targets are rare, with only 91 incidents in a 50-year period. None of the attacks resulted in radioactive fallout or environmental contamination. Most of the attacks took place outside a nuclear power plant.

## Introduction

In August 2022, the United Nations expressed its concern about the possibility of a nuclear disaster, as the Zaporizhzhia nuclear power plant in the city of Enerhodar in the Southeast of Ukraine along the left bank of the River Dnipro was once again shelled. China, the United States, and the United Nations Secretary General Guterres all warned that a safe perimeter of demilitarization to ensure the safety of the area was urgently needed.^1^

State-sponsored terrorism is not accounted for in the Global Terrorism Database (GTD). As such, these incidents will not be listed in this analysis of 50 years of terrorism.^2^

Nuclear facilities, nuclear scientists, and nuclear transport have been targets of terrorism for a long time. Police investigations into the Belgian Islamic State of Iraq and the Levant (ISIL)-inspired faction responsible for the 2015 Paris attacks and the 2016 Brussels attacks revealed that the assailants were collecting intelligence on a nuclear research plant in Mol, Belgium. They shadowed a senior director of the nuclear plant, probably in preparation for an attempted kidnapping. Whether they tried to pass security and steal nuclear material to create a dirty bomb or planned a bombing on the plant site remains unknown.^3,4^

A recent GTD study on terrorist attacks with the use of chemical, biological, radiation, and nuclear (CBRN) agents found that the use of these agents accounted for less than 0.3% of all terrorist incidents. Only 12 radiation attacks and no nuclear attacks were listed in this series, which covered five decades of terrorism.^5^

Thus far, studies of terrorist attacks against nuclear facilities, scientists, and transport have not been reported. The objective of this study was to identify and characterize all documented terrorist attacks against nuclear transport, nuclear facilities, and nuclear scientists reported to the GTD over a 50-year period. The differences in terrorist faction type, target type, and weapon type used were analyzed and the economic costs of the incidents were assessed.

## Methods

A database search of the GTD was performed by using the Preferred Reporting Items for Systematic Reviews and Meta-Analyses (PRISMA) standard.^6^

The GTD is an open-source database containing over 200,000 global terrorism incidents that occurred in the period from 1970-2020. The GTD is maintained by the National Consortium for the Study of Terrorism and Responses to Terrorism (START) at the University of Maryland (College Park, Maryland USA) and is part of the US Department of Homeland Security (Washington, DC USA) Center of Excellence.^2,7^

The GTD defines a terrorist attack as follows: *“the threatened or actual use of illegal force and violence by a non-state actor to attain a political, economic, religious, or social goal through fear, coercion, or intimidation.”* ^2^ To be considered for inclusion in the GTD, the following three attributes must all be present:

1. The incident must be intentional;
2. The incident must entail some level of violence or immediate threat of violence; and
3. The perpetrators of the incidents must be subnational.

Additionally, to be included in the database, two out of three of the following criteria must be present:

1. The act must be aimed at attaining a political, economic, religious, or social goal;
2. There must be evidence of an intention to coerce, intimidate, or convey some other message to a larger audience than the immediate victims; and/or
3. The action must be outside the context of legitimate warfare activities.

An extensive description of their origin and the data collection methodology can be found in the GTD codebook, which is available on the START website.^2,7^

The full dataset of the GTD was searched for terrorist attacks against nuclear facilities, nuclear scientists, nuclear transport, and other nuclear industry-related targets. Due to loss of data, incidents from 1993 are not present in the online database. The following search terms were applied in the database: “nuclear,” “radioactive,” “cesium,” “radioisotopes,” “plutonium,” “radium,” “radionuclides,” “polonium,” and “uranium.” Incidents were included if the aim of the attack was to target a nuclear plant, a site under construction, nuclear scientists, a nuclear research laboratory, construction firms active on a nuclear plant, nuclear transports, and uranium mines and mining companies.

Duplicates were excluded. Each attack involved in coordinated attacks is listed separately in the GTD. In this study, these were listed as one attack and used the total number of fatalities/injured.

Cases in which there was insufficient information to determine whether a nuclear target was involved were further explored using reviews of gray literature. If information remained insufficient, the cases were subsequently excluded. Last, incidents coded as “Doubt Terrorism Proper” were also excluded. These are incidents in which there was doubt if they were exclusively terrorism.^7^

Data collected per incident included temporal and spatial factors, location (country, world region), type of target, attack and weapon type, perpetrator type, number of casualties, and value of property damage.

Each entry was reviewed manually by the lead researcher for inclusion or exclusion based on the incident description. A second author (DB) reviewed each entry, as well as the excluded incidents. In case of doubt or discrepancies, a third author (FS) advised on the final decision. All collected data were exported into Excel spreadsheets (Microsoft Corporation; Redmond, Washington USA) and analyzed descriptively. Chi-squared tests were applied to evaluate the trends of incidents over time and the differences in casualties, conducted with a significance level of P <.05.

## Results

**Figure 1.**
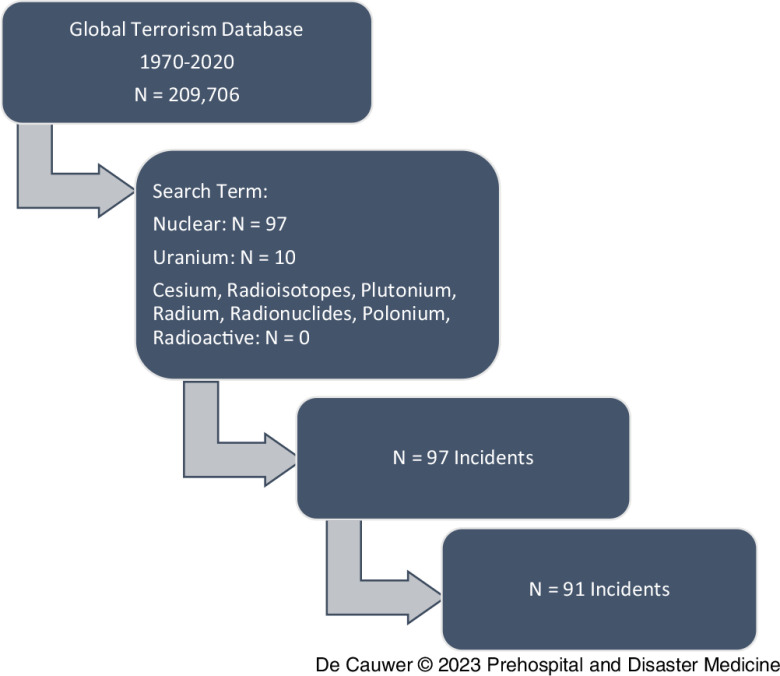
PRISMA Diagram: Step 1 – *Identification* of All Registered Incidents in the GTD; Step 2 – *Screening* for Incidents with Search Terms; Step 3 – *Eligibility*, N = 10 Incidents Excluded because of Doubt of Nuclear Target; Step 4 – Final *Inclusion* with N = 6 Duplicates Excluded.

From 1970 through 2020, the GTD contained 91 incidents against nuclear targets that fulfilled the inclusion criteria (Figure 1).

The search term “nuclear” retrieved 97 results, while “uranium” retrieved ten. There were no hits for “cesium,” “radioisotopes,” “plutonium,” “radium,” “radionuclides,” “polonium,” and “radioactive.”

Some incidents were excluded because no clear nuclear target was mentioned in the description.

The assassination of Enrique Casas, the Senator main socialist candidate for the Spanish elections in 1984, but also a nuclear physicist, was not included in this series because of the primarily political motives.

In December 1982, eight tourists were held hostage at the Washington Monument (National Mall, Washington, DC USA) by a nuclear arms protester. This incident was also excluded from this series because it did not meet the inclusion criteria.

After omitting another six duplicate incidents, a final total of 91 incidents were included in this series.

### Events per Year and Decade and Number of Victims

**Figure 2.**
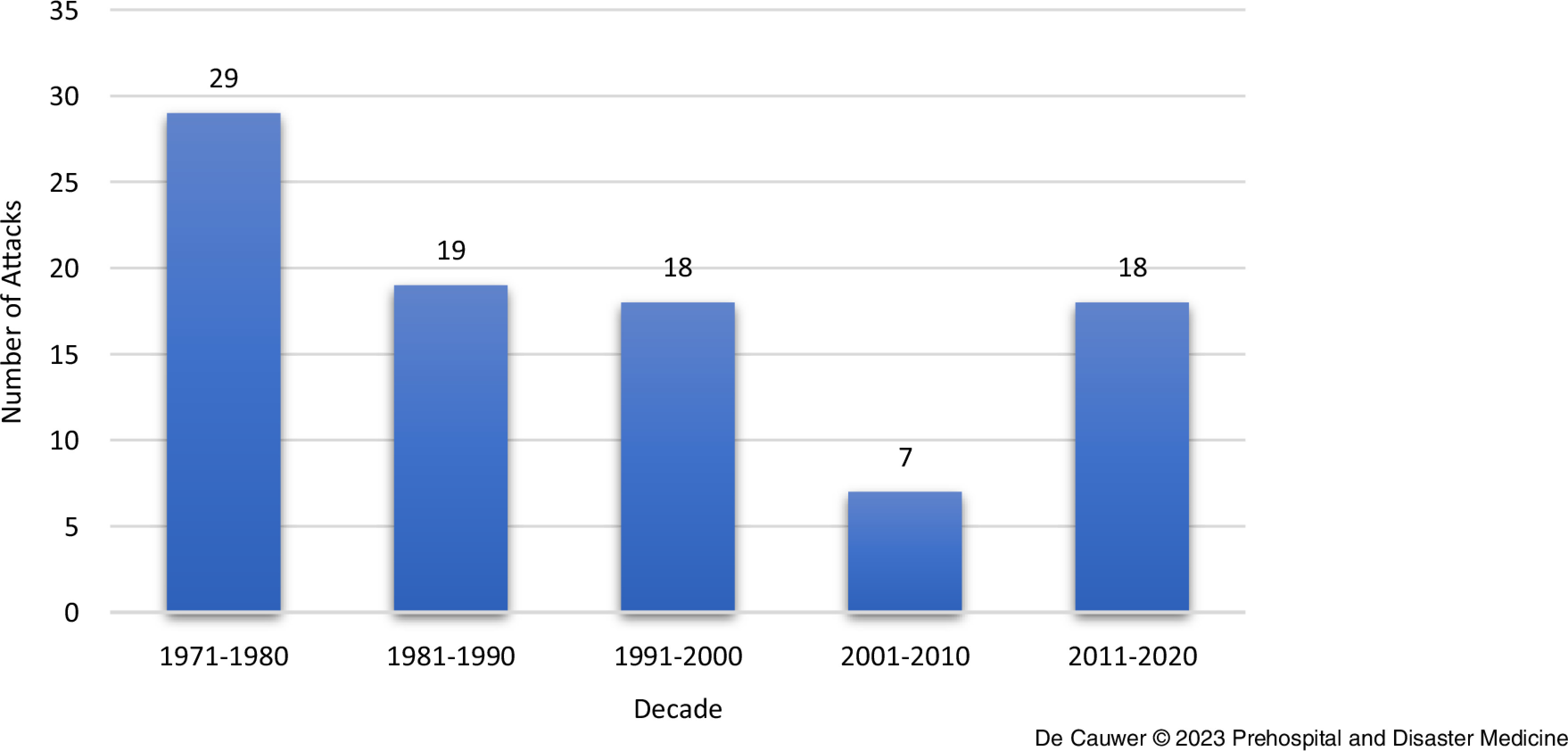
Number of Attacks per Decade from 1971 through 2020. Note: There were no incidents in 1970.

Figure 2 and Table 1 depict the number of terrorist attacks per decade.

**Table 1.** Number of Attacks and Victims per Decade

Decade	Number of Attacks	People Killed	People Injured	Perpetrators Killed	Perpetrators Injured
1971-1980	29	10	67	0	0
1981-1990	19	2	18	0	0
1991-2000	18	2	2	0	1
2001-2010	7	1	0	1	0
2011-2020	18	4	30	0	0
**Total**	**91**	**19**	**117**	**1**	**1**

Most incidents occurred in the first three decades of this series. From 2001 through 2010, the number of incidents was relatively low (n = 7), but it returned to the long-term average in the final decade.

A chi-square test to evaluate the difference in the number of attacks per decade showed a significant difference in the number of attacks: *X* ^2^ = 74.7838; *P* <.00001 (Appendix A; available online only).

During the 50 years of this analysis, 91 incidents resulted in 19 fatalities and 117 injuries. One perpetrator was killed during an incident and one other assailant was injured.

Most injuries occurred in 2003 when protestors turned violent in Buan, South Korea wounding 60 police officers and protestors. The protest was in response to a planned nuclear waste dump in the area.

### Events per Region

**Figure 3.**
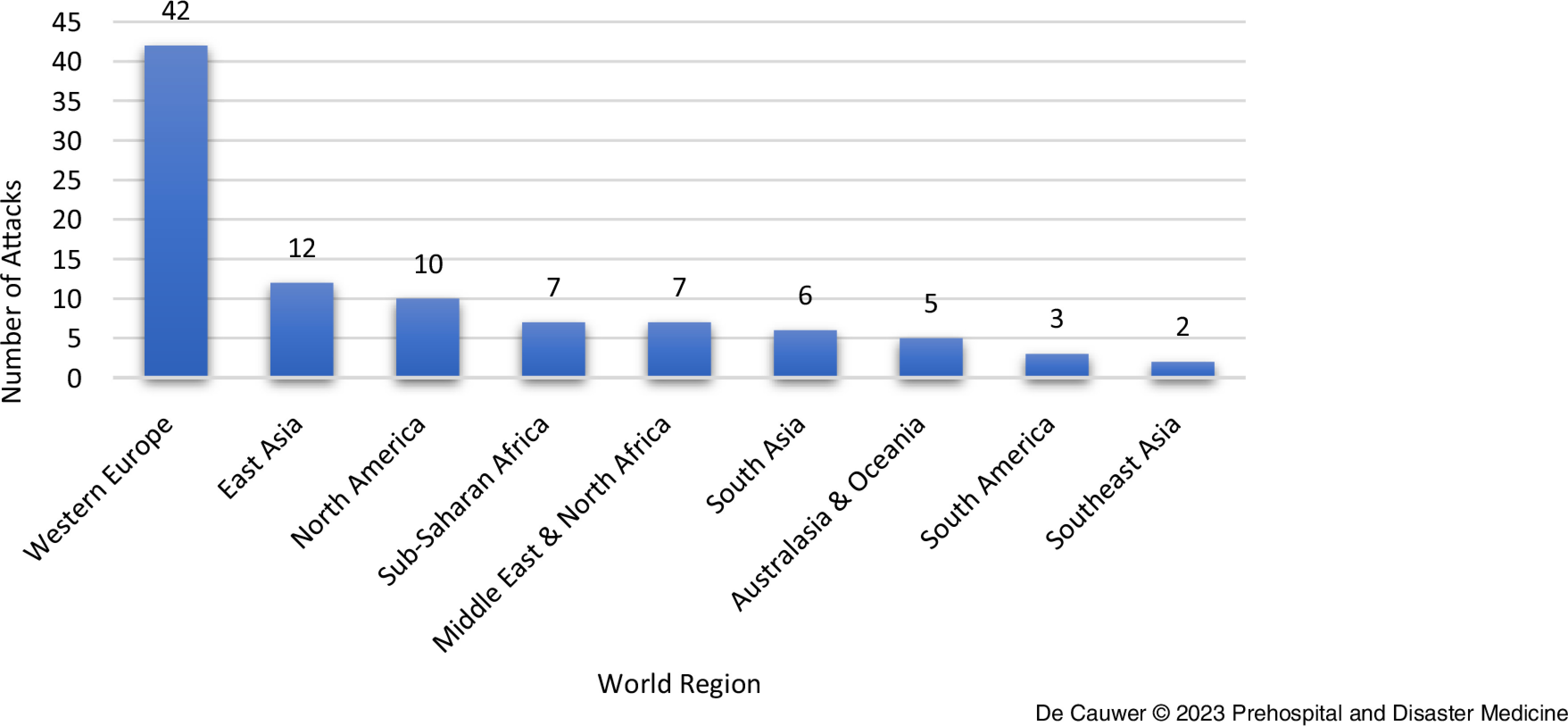
Distribution of Attacks per World Region.

Incidents took place in 25 countries and nine world regions. With 42 (46.1%) out of 91 attacks, the most frequently affected world region was Western Europe (Figure 3). East Asia ranked second with 12 (13.2%) attacks, followed by North America with 10 (11.0%) attacks.

**Figure 4.**
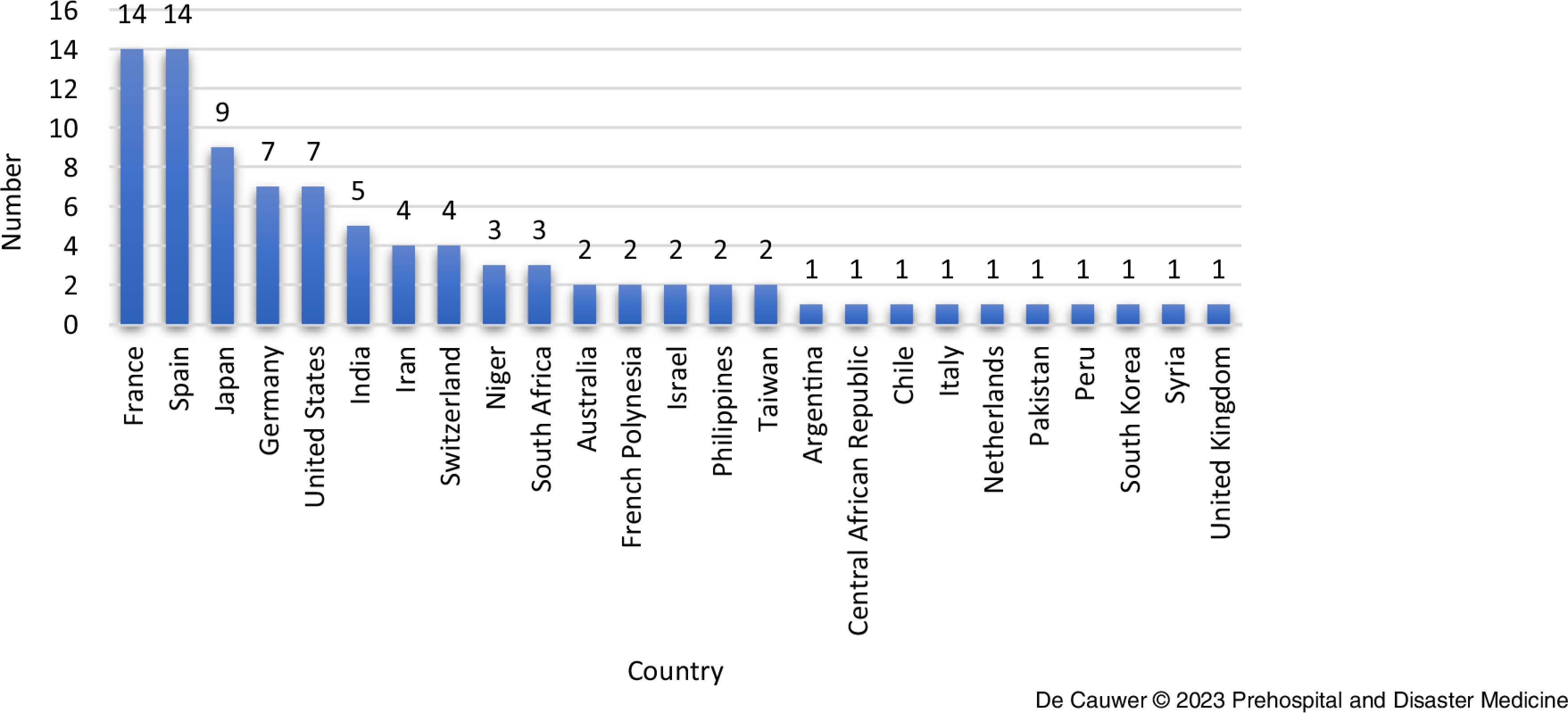
Distribution of Attacks per Country.

France and Spain (both n = 14, 15.4%) were the most commonly affected countries (Figure 4).

### Attack Types and Weapon Types

Bombings and explosions were the most frequently identified attack type (n = 40; 44.0%), followed by facility/infrastructure damage (n = 24; 26.4%) and armed assaults and assassinations (both n = 7; 7.7%); Table 2.

**Table 2.** Number of Registered Casualties per Attack Type during Incidents against Nuclear Targets, 1970-2020

Attack Type	Incidents (n)	People Killed (n)	People Injured (n)
Bombing/Explosion	40	6	30
Facility/Infrastructure Attack	24	0	13
Armed Assault	7	6	61
Assassination	7	4	3
Hostage Taking	5	1	1
Unarmed Assault	1	0	0
Hijacking	1	0	0
Mixed	2	1	4
Unknown	4	1	5
**Total**	**91**	**19**	**117**

The predominant weapon types were explosives (n = 40; 44.0%), incendiary (n = 18; 19.8%), and firearms (n = 11; 12.1%); Figure 4. Other weapon types were uncommon (Table 3). Table 2 and Table 3 show the number of victims per attack type and weapon type.

**Table 3.** Number of Registered Casualties per Weapon Type during Incidents against Nuclear Targets, 1970-2020

Weapon Type	Incidents (n)	People Killed (n)	People Injured (n)
Explosives	40	8	32
Incendiary	18	0	13
Firearms	11	7	3
Sabotage Equipment	7	0	0
Mixed Weapons	7	1	64
Unknown	5	2	5
Melee	1	0	0
Vehicle	1	1	0
Fake Weapons	1	0	0
**Total**	**91**	**19**	**117**

Bombings/explosions and armed assaults were responsible for most of the fatalities. The previously mentioned South Korean incident caused most of the injured in this series. Bombings/explosions rank second, followed by facility/infrastructure attacks.

**Figure 5.**
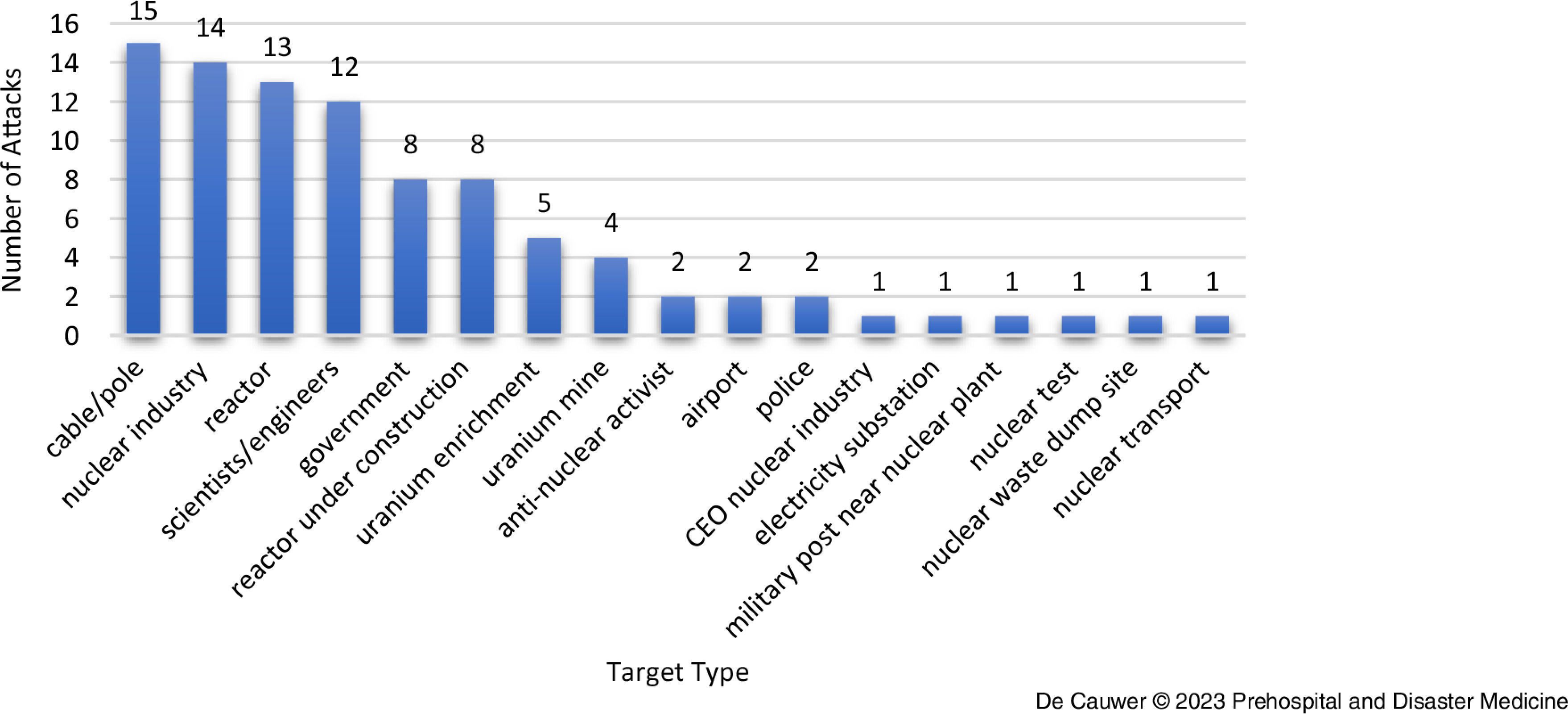
Number of Incidents per Target Type.

The target profile was diverse, as very different factions were active (Figure 5).

Nuclear power plants (reactor) were targeted in 13 (14.3%) incidents, being the only incidents that possibly could lead to a nuclear reactor containment breach. Reactors under construction were targeted in eight (8.8%) incidents. Most of the attacks took place outside a nuclear power plant: incendiaries or sabotage of cables/poles were predominant (n = 15; 16.5%), followed by attacks against the nuclear industry involved in the nuclear chain (n = 14; 15.4%), and attacks against nuclear scientists (mostly assassinations) or engineers (n = 12; 13.2%). Attacks aimed at uranium enrichment facilities and uranium mines were less frequent.

### Perpetrators and Number of Casualties per Perpetrator Type

Various perpetrator groups and concomitant motives were mentioned in the GTD. Anti-nuclear extremists were predominant in this series, followed by separatist factions (Table 4).

**Table 4.** Characteristics of Perpetrator Type and the Number of Victims per Perpetrator Group Type

Perpetrator Type	Number of Attacks	People Killed (n)	People Injured (n)
Anti-Nuclear Extremist	39	1	14
Unknown	22	4	63
Separatist	21	8	25
Jihadists	4	6	14
Left Wing	4	0	0
Anarchists	1	0	1
**Total**	**91**	**19**	**117**

With some perpetrators, the motives and any relation to existing terrorist factions could not be demonstrated, while in other attacks, no one claimed responsibility.

Lone actor attacks occurred eight times. Seven incidents were caused by the same man in 2015 who stated that he targeted East Japan Railway Company because the company used excessive electricity.

Separatist factions and jihadists caused the most fatalities (Table 4).

**Figure 6.**
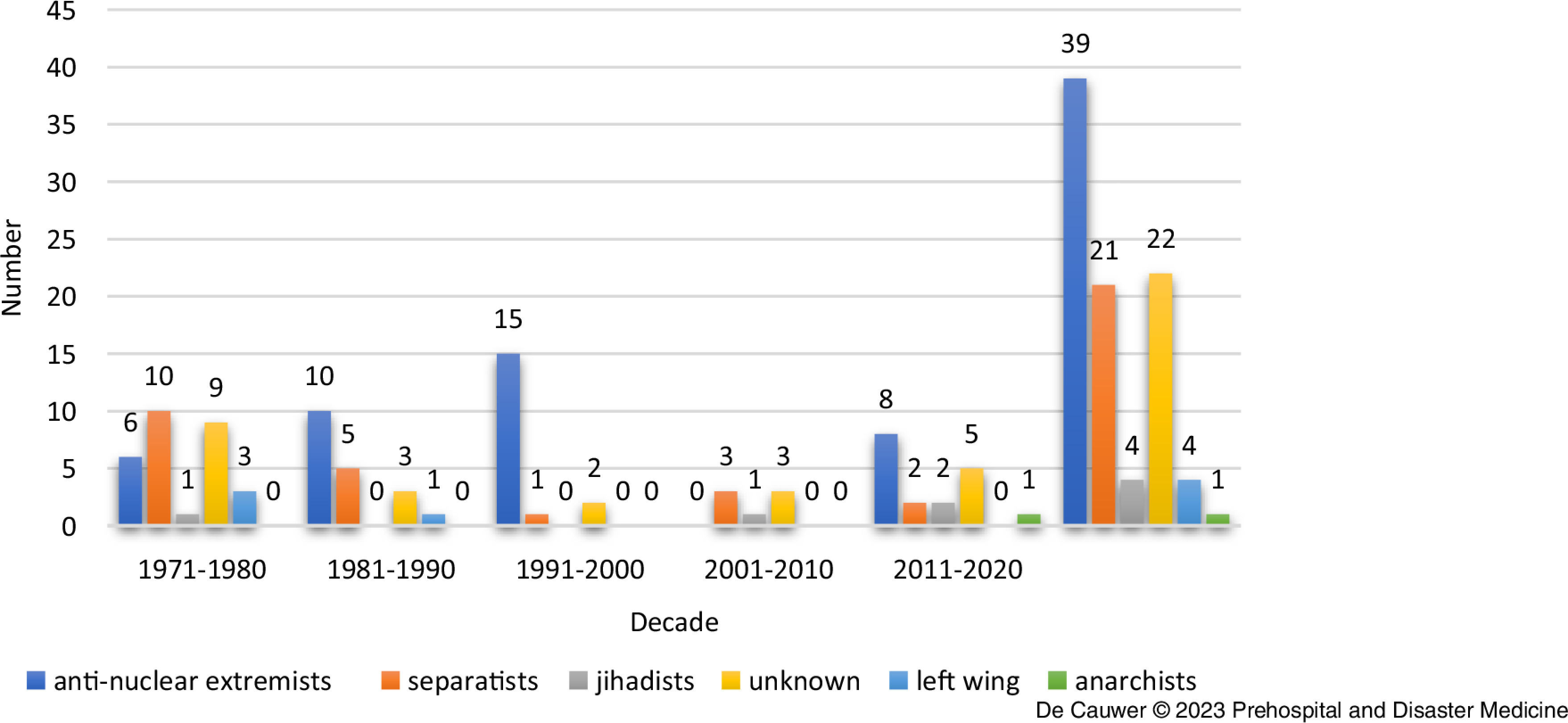
Number of Attacks per Perpetrator Type and per Decade.

During the 50-year registration period, some shifts were noted in the perpetrator type profile (Figure 6).

During the first three decades, there was a gradual increase in the number of attacks from anti-nuclear extremists. In the period from 2001-2010, these factions were not active at all, and after 2011, there was a new uptick from anti-nuclear groups. Left-wing factions were only active during the first two decades. Separatist groups dominated the first two decades, while fewer attacks were attributed to them during the last decades.

### Property Value Losses

For less than one-half of the incidents, the economic cost and property value loss were mentioned in the GTD files.

Ten incidents were not associated with economic loss or property damage. In 21 incidents, the loss was less than one million USD. In three incidents, higher economic loss was mentioned (1.2, 2.5, and 5.6 million USD).

## Discussion

This study demonstrates that diverse terrorist factions are responsible for terrorist attacks against nuclear industry-related targets. Separatist factions and jihadists caused the most fatalities, while anti-nuclear extremists (also driven by nuclear waste and environmental concerns) rather aimed at installations and construction sites.

None of the GTD-listed attacks resulted in a nuclear reactor containment breach, radioactive fallout, or environmental contamination. Nevertheless, the risk for such a major incident is real. A nuclear accident can also be caused by destroying offsite power and backup generators, or by destroying cooling systems. In fact, that was the case for the Fukushima disaster (2011) and what is feared will happen in the Zaporizhzhia nuclear power plant in the Southeast of Ukraine due to the Russo-Ukrainian war (2022).^1,8^

Aum Shrinkyo, Al Quada, Chechen rebels, and other factions have considered nuclear plant sabotage.^8^

In none of the GTD-listed incidents, the involvement of an insider assailant was mentioned. Nevertheless, recently insider threats gained growing interest and are considered a major component missing in long-term strategy to reduce the risks of nuclear terrorism.^9,10^

In fact, a police probe into the sabotage of a Belgian nuclear power plant in 2014 found that long before, a contractor (who left to fight in Syria in 2012 and was later convicted as part of ‘Sharia4Belgium’ terrorist group) had access to the vital area in the nuclear plant.^8^

Among the lessons learned, three are particularly important. First, do not assume that serious insider problems are not in the organization. Second, do not assume that background checks will solve the insider problem: these programs are effective but they are not bulletproof. Finally, do not assume that employees comply with security rules.^9^

Considering this, in 2020, the International Atomic Energy Agency (IAEA; Vienna, Austria) Information Circular 908 (INFCIRC/908), “Joint Statement on Mitigating Insider Threats,” dealt with two major focus areas: (1) commitment to support the IAEA to develop and implement an advanced, practitioner-level training course on insider threat mitigation; and (2) implementation of measures to mitigate insider risk by taking a risk-informed graded approach.^10^

Another concern for nuclear security specialists is the growing threat for nuclear transportation. In this series, only one such incident was listed. Such attacks would have a smaller impact, although a radiation release as a transport moved through an urban area could cause major fear and disruption.^8^

Bunn and Schlesinger concluded that even a small probability of the worst cases is enough to justify focused action to reduce the risk.^8^

The possible consequences of major radiation incidents on society and on health care systems are extensive. The nuclear power plant accidents of Three Mile Island (1979), Chernobyl (1986), and Fukushima (2011) prompted the evacuation of multiple hospitals in the contaminated areas.^11^ The strain of a Fukushima or Chernobyl-like event would have a devastating effect on a large area, including forced relocation of large populations, impaired health care, and a financial catastrophe for the electric power company.^8^ Chernobyl was a (accidental) dirty bomb. As an example of such, it is conceivable that an intentional nuclear incident would have a similar effect. Extrapolating to nuclear power stations today, in the Chernobyl disaster, 134 people developed acute radiation syndrome (ARS) due to the direct exposure to radiation. There were 28 short-term deaths, of which 95% occurred at whole body doses in excess of 6.5Gy. By the end of 2001, an additional 14 ARS survivors died from various causes.^12,13^ Moreover, this kind of disaster can result in wide-spread long-term health effects, at a long distance of the event site. Meteorological conditions made that radiological fallout from Chernobyl fell over most of Eastern/Central Europe and a lot of Western Russia. This way, radiation exposure affected residents of countries well beyond Ukraine and Belarus. A Belgian (the distance between Brussels, the capital of Belgium, and Chernobyl is 2074km) study revealed that, over 30 years, there had been a persistent higher incidence of papillary thyroid cancer among children below the age of 15 years at the time of the Chernobyl accident.^14^ Similarly, after the Fukushima accident, the average radiation dose-rates in the 59 municipalities of the Fukushima prefecture in June 2011 and the corresponding thyroid cancer detection rates in the period October 2011 to March 2016 showed statistically significant relationships.^15^ However, the SHAMISEN Consortium recently did not recommend mass or population-based thyroid cancer screening, as the negative psychological and physical effects are likely to outweigh any possible benefit in affected populations.^16^

Nevertheless, terrorists find the idea of nuclear terror strikes attractive, and that for a simple reason: such an attack would spread horror far beyond its physical effect. The most important effect would be people’s fear of contamination and radiation, which could cause mass disruption and panic.^8,17^

So-called perception-based impacts can persist, many years after a radiation incident, with reduced willingness to purchase goods/services, to invest, and to work in that region.^9^

Misperception of radiation incidents does not only involve the lay public. The willingness of emergency health care providers to unconditionally respond to disasters and emergencies was the lowest in nuclear incidents (24.88%), whereas 61.97% had no problem to respond to a natural disaster.^18^

Finally, hospitals and emergency health care services should focus more on CBRN preparedness, as a 2014 study demonstrated. Governments should provide more financial resources for hospital preparedness, as the survey revealed that under-funding hospitals was a major obstacle in realizing preparedness programs.^19^

## Limitations

The GTD is the most comprehensive, up-to-date, open access, and reliable database of terrorist incidents.^2,7^ The database, and therefore this study, is subject to several limitations. The data of events in the earlier decades are not complete. It is acknowledged by the GTD that in at least the first-half of the dataset, and in particular in the period from 1970 through 1989, the number of terrorist incidents is probably under-reported.^2,7^ The rise in the number of attacks since 2000 could be partly explained by this, but thus not account for the lower number in the 1990s. The loss of data in 1993 may also play a minor role.

Furthermore, the GTD relies on media publications for their information. Only high-quality sources are used. This creates a possible selection bias, and is no guarantee as what the validity of the database information is concerned.^2,7^ Casualty numbers conflict across sources. Following the GTD protocol, the most recent reliable estimates are reported and used in this study.

Trends over time should be interpreted with caution because of these limitations.^2^ Conversely, the GTD is a key source for global data on terrorism incidents and is the best available database of its kind. It is evaluated as the most complete record of terrorist attacks in recent decades.

Attempted but unsuccessful attacks are included in the GTD. However, threats, conspiracies, or the planning of attacks are not. The perpetrators literally had to be “out the door” to be included as an incident.

Additionally, state terrorism shows an increase in recent years but is not listed in the GTD.^2,7^ However, historic state-driven attacks on nuclear compounds demonstrate the vulnerability of nuclear reactors during war.^20^

## Conclusion

Terrorist attacks carried out by non-state perpetrators against nuclear facilities, nuclear scientists, and nuclear transport, or other nuclear industry-related targets, are rare with only 91 incidents in a 50-year period. None of the attacks resulted in a nuclear reactor containment breach, radioactive fallout, or environmental contamination. Most of the attacks took place on other nuclear industry-related sites.

## Supplementary material

For supplementary material accompanying this paper visit https://doi.org/10.1017/S1049023X2300002X. click here to view supplementary material
