# Supplementary material for: 50 Years of Terrorism against the Nuclear Industry: A Review of 91 Incidents in the Global Terrorism Database
Source: Prehosp Disaster Med. 2023 Jan 17;38(2):199–206. doi: 10.1017/S1049023X2300002X (PMC10067068; doi:10.1017/S1049023X2300002X)
Supplement: Supplementary file 1 [file S1049023X2300002Xsup001.docx]

**Appendix A.** Number of Nuclear-Related Incidents per Decade, 1970-2020.

|  | Number of Incidents | |  |  |
| --- | --- | --- | --- | --- |
|  | Observed | Expected | Chi-Square Value | *P Value* |
| 1971 – 1980 | 29 | 18.2 | 74.7838 | P <.00001 |
| 1981 – 1990 | 19 | 18.2 |  |  |
| 1991 – 2000 | 18 | 18.2 |  |  |
| 2001 – 2010 | 7 | 18.2 |  |  |
| 2011 – 2020 | 18 | 18.2 |  |  |
| Total Number of Incidents | 91 | 91 |  |  |

The result is significant at P <.05.
